# Three-Dimensional Evaluation of Dental Arches in Individuals with Syndromic Craniosynostosis

**DOI:** 10.1155/2023/1043369

**Published:** 2023-01-07

**Authors:** Rayane de Oliveira Pinto, Cristiano Tonello, Adriano Porto Peixoto, Adriana Souza de Jesus, Ary dos Santos-Pinto, Dirceu Barnabé Raveli

**Affiliations:** ^1^Department of Orthodontics, São Paulo State University (UNESP), School of Dentistry, Araraquara, Brazil; ^2^Craniofacial Department, Hospital for Rehabilitation of Craniofacial Anomalies, University of São Paulo, Bauru, Brazil; ^3^Department of Orthodontics, Hospital for Rehabilitation of Craniofacial Anomalies, University of São Paulo, Bauru, Brazil

## Abstract

**Objective:**

Individuals with syndromic craniosynostosis present alterations in the dental arches due to anomalies caused by the early fusion of the craniomaxillary sutures. This study aimed to compare intradental and interdental dimensions between individuals with Apert and Crouzon syndromes and nonsyndromic controls.

**Materials and Methods:**

Digital models were obtained from the archive of a public tertiary care hospital. The sample consisted of 34 patients (Apert *n* = 18, Crouzon *n* = 16) and 34 nonsyndromic controls matched for gender and age. Measurements of perimeter, length, intercanine and intermolar distances (upper and lower), overjet, and molar ratio were performed. Statistical comparisons were performed using ANOVA and Tukey tests (*p* < 0.05).

**Results:**

Patients with Apert and Crouzon syndromes have severely reduced maxillary transverse dimensions, perimeter, and length of the upper arch compared to the control group (*p* < 0.001). The lower arch is less impacted. Patients with Apert syndrome had an anterior crossbite (*p* < 0.001), while patients with Crouzon syndrome had an edge-to-edge bite (*p* <  0.011). Patients with Apert and Crouzon syndromes do not have serious transverse proportion problems when comparing the upper and lower arches.

**Conclusions:**

In this sample, both the Apert and Crouzon groups have severely compromised upper arches compared to the control group. Mild dentoalveolar expansion in the maxilla should be sufficient for the transverse adaptation of the dental arches before frontofacial advancement.

## 1. Introduction

Craniosynostosis is fundamentally characterized by the early closure of cranial sutures, which can be found in isolation or associated with syndromes, being called syndromic craniosynostosis (SC). SC occurs at an incidence of 1 in 30,000-1 : 100.00 babies born alive [1], and among the most common manifestations are the syndromes of Apert (OMIM 101200) [2] and Crouzon (OMIM 123500) [3]. These syndromes are part of the group of autosomal dominant congenital anomalies caused by mutations in the fibroblast growth factor receptor found in the FRFR2 or TWIST1 gene [4].

Individuals with SC are affected by severe midfacial retrusion, exorbitism, orbital anomalies, respiratory compromise, and anomalies in the cranial shape and extremities [4–6]. Recent studies have shown that the retrusion of the middle face in Crouzon (CS) and Apert (AS) syndromes is due to dysmorphology of the sphenoid bone and maxilla, which have reduced dimensions in the transverse and anteroposterior directions [7].

Maxillary growth alterations represent a strong genetic etiology in the development of malocclusions such as Class III dental relationship, negative tooth-bone discrepancy, and impacted and ectopic teeth [8–10]. These alterations are detected in both arches, but they are more severe in the upper arch [11]. Muscle changes such as low tongue posture and mouth breathing are also contributing factors to maxillary underdevelopment [12].

Individuals with CS and AS have differences in craniofacial growth and intraoral characteristics. Patients with Apert syndrome have increased frontal facial height and a severely decreased middle third of the face, clockwise rotation of the jaw, and anterior open bite. Patients with Crouzon syndrome do not express changes in the lower third or anterior facial height of the face. They present a reduction in the middle third of the face and passive lip sealing [6].

Rehabilitative treatment of the SC patient includes intracranial decompression surgery and tracheostomy, if necessary, soon after birth. At 6–12 months of age, the first frontofacial advancement is performed, and syndactyly correction is indicated in patients with AS. Ocular correction surgeries and frontofacial advancement. From 1 to 2 years of age, palatoplasty is performed or planned, if necessary. From 2 to 7 years of age, dentition development is monitored and followed by the multidisciplinary team. From the age of 7–9 years, the patient is referred for the first consultation with the orthodontist to perform tooth extractions to correct crowding and indication of maxillary expansion to prepare for the frontofacial advancement, Le Fort III [13, 14].

Recent findings by Meazzini et al. [15] evidence early fusion of circumaxillary sutures in patients with Apert, Crouzon, and Pffeifer syndromes. The study evaluated the pterygomaxillary, midpalatal, and zygomaticomaxillary sutures through computed tomography. The group of syndromic individuals presented early ossification in all evaluated structures, compared to the control group.

Considering the need for orthodontic management in the rehabilitation process of patients with SC and the limitations imposed by craniomaxillary anomalies, detailed knowledge of dental arches is of paramount importance. The objective of this work was to evaluate the level of atresia of the upper arch in relation to the lower arch, molar ratio, overjet, perimeter, and arch length through linear measurements in digital models, comparing them with their respective controls.

## 2. Materials and Methods

This retrospective longitudinal case-control study was carried out with the approval of the Ethics and Research Committee of the Hospital for Rehabilitation of Craniofacial Anomalies, University of São Paulo (HRAC-USP) (Protocol 31741120.7.0000.5441). The sample consisted of 34 digital models of CS patients, 18 for the Apert syndrome group and 16 for the Crouzon syndrome. Among the controls, 34 digital models of Class I patients were selected, with a maximum of ¼ of Class II or Class III, matched for gender and age. Patients of both genders, aged 5 to 26 years, were included in the study.

The patients were selected from a secondary database of patients with CS previously treated by the craniofacial team at HRAC-USP. The control group consisted of a sample of 34 digital study models of nonsyndromic individuals, matched for sex and age with the syndromic individuals (Crouzon and Apert syndromes), seen at the private clinic of one of the authors. Subsequently, the patients were subdivided according to the stage of dentition development (mixed or permanent) for comparison purposes (Table 1).

The inclusion criteria were patients with a clinical and genetic diagnosis of Crouzon or Apert syndrome, performed by specialists from the craniofacial team (geneticist, craniofacial surgeon, neurosurgeon, speech therapist, and orthodontist). All medical records were reviewed for consultation on previous dental procedures. The models selected in this study were performed using molding before any orthodontic treatment or secondary craniofacial surgery. Cases with inferior quality digital models were excluded, as were cases of missing teeth that made it impossible to measure the dental arches provided for in the methodology.

After digitizing the sample models and obtaining the STL files (Stereolithography), eleven validated measurements were performed. The models were previously oriented in the 3 planes using 3D Slicer software (NA-MIC, USA).

**Figure 1:**
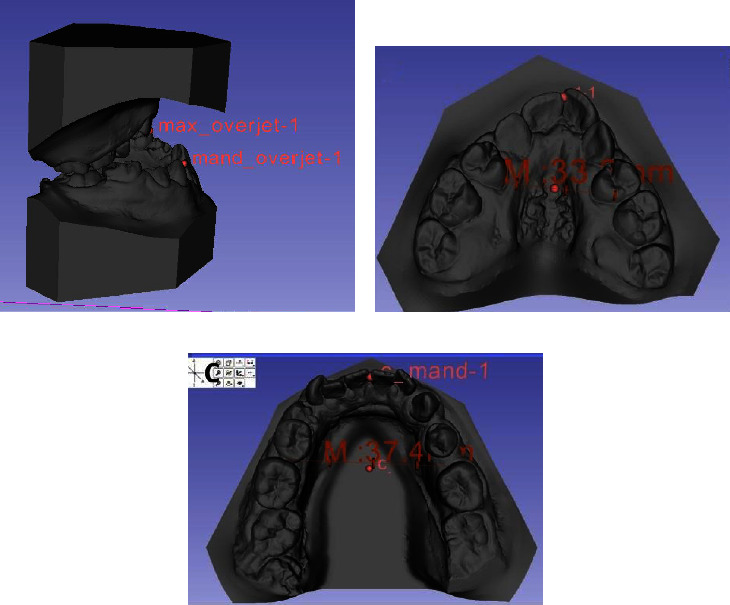
Measurements performed in the 3d slicer software. (a) Overjet. (b) Length of the upper arch. (c) Length of the lower arch.

**Figure 2:**
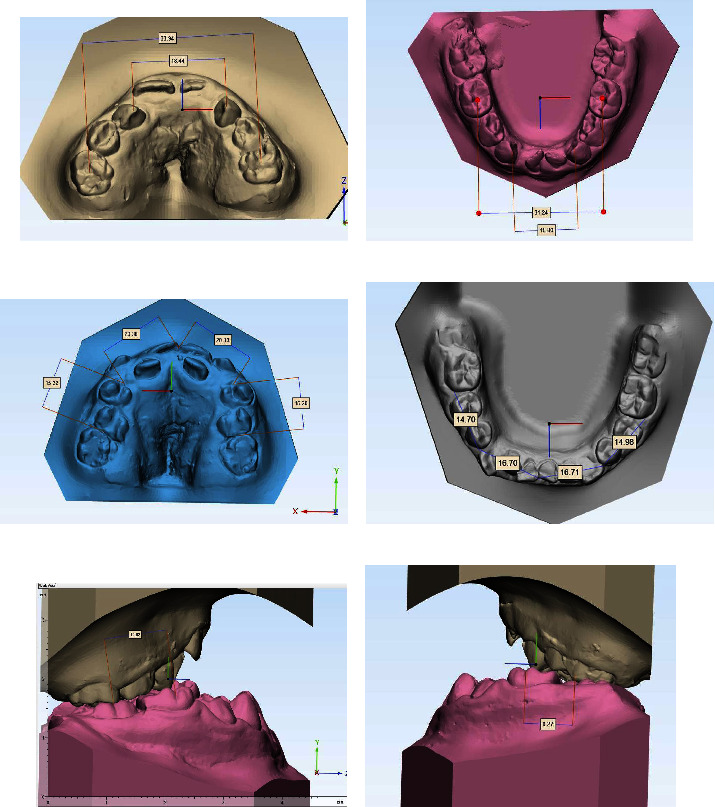
Measurements performed in the 3-matic software. (a) Intercanine and intermolar distances in the upper arch. (b) Intercanine and intermolar distances in the lower arch. (c) Perimeter of the upper arch. (d) Perimeter of the lower arch. (e) Right side molar relationship. (f) Left side molar relationship.

In this software, measurements of overjet and upper and lower arch length were performed. In the 3-matic software (Materialise, Leuven, Belgium), measurements were taken of the molar ratio of the right and left sides, perimeter, intercanine and intermolar distances in the upper and lower arches (Figures 1 and 2).

Overjet: the anteroposterior distance between the buccal surface of the lower incisor and the incisal edge of the upper incisor.

Molar relationship: the anteroposterior distance between the mesiobuccal cusp of the upper first molar and the central sulcus of the lower first molar.

Arches perimeter: measured from the mesial surface of the first permanent molars, following the contour of the arches on the contact points and the incisal edges in a smooth curve to the mesial surface of the first permanent contralateral molar.

Arches length: the anteroposterior distance between the palatal surface of the central incisor, passing through the center of the arch until finding the imaginary line that passes through the mesial surface of the first permanent molars.

Intercanine distance: measured from the canine cusp tip on one side of the arch to the contralateral canine.

Intermolar distance: measured between the central pits or estimated central pits (if there are restorations or fissure sealants) of the first permanent molars.

In cases where the first permanent molar was absent, the second primary molar was used as a posterior reference. In cases where permanent canines were absent, the buccal cusp of the first premolars was used as a reference. Mixed dentition cases in which primary canines were absent were excluded from the sample.

All variables were obtained and analyzed by 1 calibrated examiner, in both control and study groups.

### 2.1. Statistical Analysis

After 15 days, the measurements of 15 models drawn from the 4 groups were repeated to obtain the intraclass correlation index (ICC). A correlation coefficient of 0.75 or more was considered to have a high degree of reliability. A descriptive analysis was used to characterize the study population (Table 1). The characteristics of the dental arch of individuals in the Apert, Crouzon, and control groups were compared using the ANOVA test. Fisher's one-way analysis of variance was performed for the variables in the analysis.

To identify differences between groups, Tukey's post hoc analysis was performed. In all tests, a significance level of 5% (*p* < 0.05) was adopted. For comparison between the 3 groups and to study possible interferences in the studied variables, the univariate test was performed considering the independent variables dentition, group, and gender. All statistical procedures were performed using the SPSS version 26 program.

## 3. Results

### 3.1. Study Sample

All controls had positive overjet and overbite and no posterior crossbite. Of the 18 patients with Apert syndrome, 8 were girls and 10 were boys. Among the 16 patients with Crouzon syndrome, 10 were girls and 6 were boys. The mean age of individuals with AS was 14.4 years (SD 6.6) and with CS was 13.4 years (SD 4.0) (Table 1). Most patients with AS and CS had a Class III or Class III subdivision, *n* = 20. 11 syndromic patients had a Class II or Class II subdivision (Table 2). Regarding the anterior relationship of patients with AS or CS, 10 patients had an increased overjet and 16 had an anterior crossbite (Table 2).

### 3.2. Apert vs. Control

The results of the comparison between individuals with Apert syndrome and their respective controls are detailed in Tables 3 and 4. Patients with Apert syndrome have severely reduced upper arch perimeter and length compared to controls (*p* < 0.001). The intermolar and intercanine distances were also markedly reduced in the upper arch (*p* < 0.001). The lower arch perimeter is also markedly reduced compared to controls (*p* < 0.001). The lower arch of individuals with AS is shorter compared to the arch length of controls (*p* < 0.005). Patients with Apert syndrome had an anterior crossbite with an overjet of −3.56 mm (*p* < 0.001). The variables, intermolar and inferior intercanine distances, as well as the molar relationship of the right and left sides, did not present statistically significant differences. Apert syndrome has a markedly reduced upper arch perimeter and length compared to controls, with the significance of (*p* <  0.001) and (*p* <  0.003), respectively. Individuals with AS had Class III malocclusions on the right (*p* < 0.062) and left (*p* < 0.047) sides.

Furthermore, the complementary Table 5 shows the distribution of the variables according to group and dentition.

### 3.3. Crouzon vs. Control

The results of the comparison between individuals with Crouzon syndrome and their respective controls are detailed in Tables 3 and 4. Patients with Crouzon syndrome present the perimeter and length of the upper arch markedly reduced when compared to the controls, with significance of (*p* <  0.001) and (*p* <  0.003), respectively. Individuals with CS presented a narrowing of the intermolar distance (*p* < 0.001), but no differences were seen in comparison to the controls for the intercanine distance.

The perimeter of the lower arch in the group with CS was reduced (*p* <  0.018), while the length of the lower arch showed no changes compared to the control group. Patients with CS have reduced intercanine distance (*p* < 0.036) and intermolar distances without any alterations compared to nonsyndromic patients. Patients with CS had an edge-to-edge bite with 0 mm overjet (*p* < 0.011) and Class III malocclusion on the right (*p* < 0.062) and left (*p* < 0.047) sides.

### 3.4. Apert vs. Crouzon

The results of the comparison between individuals with Apert and Crouzon syndromes are detailed in Tables 3 and 4. Individuals with AS have a reduced upper arch perimeter compared to patients with CS (*p* < 0.001). Patients with AS had an anterior crossbite of −3.65 mm, while patients with CS had an edge-to-edge bite with an overjet of 0 mm (*p* < 0.006). The other variables evaluated did not show statistically significant differences between the groups.

Comparing the intermolar distances of the upper arch (39.89 mm) with the lower arch (39.77 mm) in patients with CS, no great differences in proportion are observed between them. In the group with AS, the same condition occurs for the upper (37.95 mm) and lower (38.57 mm) intermolar distances.

In the univariate analysis test considering dentition (mixed or permanent), gender, and group (Table 4), the patient's dentition interfered with statistical significance in the variables perimeter (*p* = 0.011) and length (*p* < 0.001) of the lower arch. Gender interfered in the variables perimeter (*p* = 0.021), length (*p* = 0.002), and intermolar distance (*p* = 0.044) of the upper arch. The group (Apert or Crouzon) interfered in all variables except inferior intercanine distance.

### 3.5. Reproducibility

The statistical analysis reliability in obtaining replicated measurements showed high reproducibility in the measurements of the study variables, with Cronbach alpha values greater than 0.992 (ICC greater than 0.990) and a mean difference not exceeding 0.6 mm. The lower arch perimeter showed a statistically significant difference in measurements, on average 0.47 mm.

## 4. Discussion

It is particularly important to have a better understanding of the changes that occur between dentitions in the maxillary and mandibular arches and features associated in both syndromic and nonsyndromic children to better develop preventive and therapeutic strategies for the syndromic patients.

The archers' measurements, in the control subjects of the current study, for dental arch widths, lengths, and overjets were comparable with those reported in the literature [16]. The results of the present study show that AS and CS patients have severely reduced maxillary dimensions (perimeter and length) compared to the control group. The intermolar distance was also markedly reduced in the upper arch for both AS and CS. As for maxillary intercanine distance, only AS showed a significant difference from the control group. In the comparison between the AS and CS groups, the intercanine distance was also significantly smaller in patients with AS.

The studies by Tonello et al. [17] and Reitsma et al. [18] showed similar results, with maxillary and mandibular dental arch lengths measured for patients with AS and CS syndromes statistically smaller than in control subjects. Reitsma et al. [18] also analyzed the growth model of these patients, and no changes over time in maxillary intermolar width for patients with CS were observed, whereas maxillary intercanine widths increased. Patients with AS showed no change over time for the maxillary intercanine width variable.

Together, these findings suggest that, transversely, the anterior region of the maxilla in patients with CS is not as affected as the posterior region and still has the potential to increase with the patient's growth. Moreover, the anterior maxillary region of CS patients is less affected than that of AS patients. This reflects the type of malocclusion observed in our sample, where patients with AS had a more anterior crossbite (mean overjet of −3.65) while patients with CS had an edge-to-edge bite (mean overjet of 0 mm). Studies on frequent findings in AS and CS patients reported anterior crossbite in more than 80% of the cases analyzed [6, 8]. A previous study, although performed with a small sample, found mean overjet values of −8.2 and −5.3 mm for AS and SC, respectively [6]. It was also previously reported that patients with AS tended to have smaller arch dimensions and more severe deformities in the craniofacial region than CS patients [6, 18].

In the current sample, the mandibular arch perimeter is also reduced compared to controls in AS and CS. However, between the AS and CS groups, there were no significant differences in mandibular measurements. The lack of maxillary growth and development caused by craniosynostosis in these syndromes can result in many occlusal problems in both arches but are more severe in the maxilla [19]. The mandible seems to be indirectly influenced by the absence of primary and secondary displacement of the bones of the maxillary complex and the base of the skull [18].

The found interferences regarding the patient's dentition in the variables perimeter (*p* = 0.011) and length (*p* < 0.001) (Table 4) of the lower arch were considered normal related to the development of occlusion in the transition of the mixed to permanent dentition. The decrease in perimeter and arch length from mixed to permanent dentition occurs because of molar mesialization at the end of the second transitional period. The dental arch length did not change in the maxilla, but it decreased slightly in the mandible, as previously reported [18].

Interestingly, this study showed that the rapid suture fusion that causes skeletal problems is not influencing so much the posterior transverse maxillomandibular relationship of these individuals. That is because AS and CS do not have serious problems in proportion when comparing the upper and lower arches of this region. The mean intermolar distances were 39.89 mm for the upper arch and 39.77 mm for the lower arch in CS group. In the group with AS, the same condition occurs for the upper (37.95 mm) and lower (38.57 mm) intermolar distances. These results indicate that mild dentoalveolar expansion in the maxilla should be sufficient for the transverse adaptation of the dental arches to treat these cases before frontofacial advancement.

Furthermore, in the analysis of the molar relationship, the most observed classification in this sample was Class III in both syndromic groups, which is corroborating with other studies' findings [20]. The prevalence of Class III malocclusion can also be attributed to an adaptation to a hypoplastic maxilla, with increased mandibular rotation and anteriorly positioned mandibular condyles [11].

Finally, due to an underdeveloped maxilla in a Class III situation, exfoliation of the maxillary deciduous canines commonly occurs during the eruption of the permanent lateral incisors, thus lacking space to accommodate the permanent canines and premolars. The need for tooth serial extractions is common to better accommodate permanent teeth [21]. Individuals with SA and SC can benefit from this approach to treating intraarches crowding.

We consider that the limitation of the current study was its retrospective design, in which a convenience sample was used and in which generalizations considering the total population should be made with caution. In addition, the sample has a wide age range. Despite this, all individuals in the syndromic groups were age-matched. Analysis of the sutures around the maxilla of these patients has been described in the literature [15]. More investigations using computed tomography analysis would allow us to better investigate the effect of the amount of fusion of the adjacent sutures in relation to the dental arch to understand the outcome of these altered dimensions.

## 5. Conclusions

- In the sample of the current article, patients with Apert and Crouzon syndromes have severely reduced transverse dimensions, perimeter, and length of the upper arch compared to the control group. The lower arch is less impacted.
- Patients with Apert and Crouzon syndromes do not have serious transverse proportion problems when comparing the upper and lower arches. Patients with Apert syndrome had anterior crossbites, while patients with Crouzon syndrome had an edge-to-edge bite.
- Mild dentoalveolar expansion in the maxilla should be sufficient for the transverse adaptation of the dental arches before frontofacial advancement.

## Figures and Tables

**Table 1:** Descriptive analysis. Age (years) by gender of patients, dentition, and study group.

Group	Gender	Mixed dentition					Permanent dentition				
		*N*	Mean	SD	Min	Max	*N*	Mean	SD	Min	Max
Apert	Female	4	9.3	2.6	7	12	4	19.8	4.8	16	26
	Male	3	6.7	2.1	5	9	7	17.6	4.6	13	26
	Both	7	8.1	2.6	5	12	11	18.4	4.5	13	26

Crouzon	Female	8	9.8	1.6	8	12	2	14.0	1.4	13	15
	Male	2	10.0	2.8	8	12	4	19.3	3.8	16	23
	Both	10	9.8	1.7	8	12	6	17.5	4.0	13	23

Control	Female	12	9.4	1.9	7	12	6	17.3	4.6	13	25
	Male	6	8.7	2.5	6	12	10	18.6	4.5	13	28
	Both	18	9.2	2.1	6	12	16	18.1	4.4	13	28

**Table 2:** Frequency of molar relationship and anterior relationship.

*Molar relationship*	*Apert*	*Crouzon*	*Control*
Class I	0	1	21
Class II	3	2	0
Class III	8	9	2
Class II subdivision	4	2	9
Class III subdivision	2	1	2
Class II and class III	1	1	0
Molar relationship of class I ±1.5 mm			

*Anterior relationship*	*Apert*	*Crouzon*	*Control*

Normal	5	3	32
Overjet	6	4	0
Crossbite	7	9	2
Normal overjet ±2 mm			

**Table 3:** Description of the variables according to the study group.

Group	Crouzon				Apert				Control			
Variable	*N*	Mean	Tukey	SD	*N*	Mean	Tukey	SD	*N*	Mean	Tukey	SD
Upper arc perimeter	16	64.15	B	7.1	18	56.44	A	7.5	34	74.92	C	3.4
Upper arc length	16	21.88	A	3.7	18	19.40	A	5.2	34	25.43	B	1.9
Upper intermolar	16	39.89	A	4.6	18	37.95	A	5.0	34	46.36	B	4.2
Upper intercanine	16	31.95	A	4.0	18	28.20	A	5.6	34	34.19	B	3.6
Lower arc perimeter	16	62.13	A	6.9	18	59.94	A	8.0	34	67.10	B	3.5
Lower arc length	16	20.41	B	3.7	18	18.90	A	4.2	34	21.84	B	1.9
Lower intermolar	16	39.77	B	4.4	18	38.57	A	5.1	34	42.10	B	2.4
Lower intercanine	16	23.95	A	3.0	18	25.32	B	4.2	34	26.29	B	2.2
Overjet	16	0.00	B	4.9	18	−3.65	A	4.4	34	3.04	C	1.2
Molar relat.right	16	−2.70		6.2	18	−2.15		5.6	34	0.26		1.2
Molar relat.left	16	−3.16	B	5.4	18	−0.67	A	6.1	34	−0.11	A	1.2

Molar relat.right: Molar relationship right, Molar relat.left: Molar relationship left

**Table 4:** Multivariate and univariate statistical tests considering group, age, dentition, and interactions.

		Group		Age group		Group✻age group	
		*F*	*P*	F	*P*	F	*P*
Multivariate tests	Pillai's trace	5.30	**<0.001**	2.04	**0.043**	1.20	0.266
	Wilks' lambda	7.88	**<0.001**	2.04	**0.043**	1.18	0.285
	Hotelling's trace	11.07	**<0.001**	2.04	**0.043**	1.15	0.306
	Roy's largest root	21.56	**<0.001**	2.04	**0.043**	1.30	0.251

Univariate tests	Upper arc perimeter	63.05	**<0.001**	0.00	0.975	0.12	0.884
	Upper arc length	18.41	**<0.001**	0.70	0.406	0.10	0.903
	Upper intermolar	25.09	**<0.001**	1.10	0.299	2.06	0.136
	Upper intercanine	11.84	**<0.001**	3.46	0.068	0.52	0.597
	Lower arc perimeter	10.81	**<0.001**	4.90	**0.031**	1.71	0.190
	Lower arc length	6.50	**0.003**	12.64	**<0.001**	2.13	0.127
	Lower intermolar	5.53	**0.006**	0.03	0.853	0.77	0.469
	Lower intercanine	3.29	**0.044**	3.11	0.083	0.28	0.758
	Overjet	24.72	**<0.001**	0.66	0.419	2.32	0.106
	Molar relat.right	3.53	**0.035**	3.72	0.058	0.44	0.645
	Molar relat.left	3.05	0.055	2.92	0.092	0.38	0.685

The values in bold were considered statistically significant. Molar relat.right: Molar relationship right, Molar relat.left: Molar relationship left

**Table 5:** Variables according to the group and dentition.

Group	Crouzon			Apert			Control		
	*N*	Mean	SD	*N*	Mean	SD	*N*	Mean	SD
Mixed dentition									
Lower arc perimeter	10	64.47	7.25	7	63.06	6.76	18	67.38	3.70
Lower arc length	10	22.11	3.21	7	20.92	4.65	18	22.38	1.68
Permanent dentition									
Lower arc perimeter	6	58.23	4.20	11	57.95	8.38	16	66.78	3.43
Lower arc length	6	17.57	2.61	11	17.61	3.59	16	21.24	2.06

## Data Availability

The data used to support the findings of this study are included within the article.
